# Aerosol Jet Printing for Neuroprosthetic Device Development

**DOI:** 10.3390/bioengineering12070707

**Published:** 2025-06-28

**Authors:** Lander De Waele, Massimo Di Pietro, Stefano Perilli, Emanuele Mantini, Giulio Trevisan, Michela Simoncini, Massimo Panella, Viviana Betti, Matteo Laffranchi, Dante Mantini

**Affiliations:** 1 Movement Control and Neuroplasticity Research Group, KU Leuven, Tervuursevest 101, 3001 Leuven, Belgium; lander.dewaele@kuleuven.be; 2 Department of Industrial Engineering and Mathematical Sciences, Polytechnic University of Marche, Via Brecce Bianche 12, 60131 Ancona, Italy; dipietro.massimo@comecinnovative.it (M.D.P.); m.simoncini@staff.univpm.it (M.S.); 3 Comec Innovative S.r.l., Via Papa Leone XIII 34, 66100 Chieti, Italy; mantini.emanuele@comecinnovative.it (E.M.); trevisan.giulio@comecinnovative.it (G.T.); 4 Department of Information Engineering, Electronics and Telecommunications, University of Rome “La Sapienza”, Via Eudossiana 18, 00184 Rome, Italy; stefano.perilli@uniroma1.it (S.P.); massimo.panella@uniroma1.it (M.P.); 5 Department of Psychology, University of Rome “La Sapienza”, Via dei Marsi 78, 00185 Rome, Italy; viviana.betti@uniroma1.it; 6 Laboratory of Neuroscience and Applied Technology, IRCCS Santa Lucia Foundation, Via Ardeatina 306, 00179 Rome, Italy; 7 Rehab Technologies Lab, Italian Institute of Technology, Via Morego 30, 16163 Genova, Italy; matteo.laffranchi@iit.it

**Keywords:** aerosol jet printing, neuroprosthetic devices, neural interfaces, additive manufacturing, biocompatible materials

## Abstract

Aerosol jet printing (AJP) technology has emerged as a transformative tool in neuroprosthetic device development, offering high accuracy and versatility in fabricating complex and miniaturized structures, which are essential for advanced neural interfaces. This review explores the fundamental principles of AJP, highlighting its unique aerosol generation and concentrated deposition mechanisms, which facilitate the use of different materials on a variety of substrates. The advantages of AJP, including its device scalability, ability to print on flexible and stretchable substrates, and compatibility with a wide range of biocompatible materials, are examined in the context of neuroprosthetic applications. Key implementations, such as the fabrication of neural interfaces, the development of microelectrode arrays, and the integration with flexible electronics, are discussed, showcasing the potential of AJP to revolutionize neuroprosthetic devices. Additionally, this review addresses the challenges of biocompatibility and technical limitations, such as the long-term stability of electroconductive traces. The review concludes with a discussion of future directions and innovations, emphasizing the realization of sensorized prosthetic limbs through the incorporation of tactile sensors, the integration of biosensors for monitoring physiological parameters, and the development of intelligent prostheses. These prospects underscore the role of AJP in the advancement of neuroprosthetic applications and its pathway toward clinical translation and commercialization.

## 1. Introduction

Neuroprosthetics is an interdisciplinary field that combines neuroscience, biomedical engineering, and materials science to design and develop devices that interact directly with the nervous system. These devices aim to restore lost sensory and motor functions in individuals with neurological disorders or injuries [1,2]. Traditional neuroprosthetic devices include cochlear implants, deep brain stimulators, and brain–computer interfaces (BCIs), which rely on increasingly miniaturized and integrated electronics to perform complex sensing, stimulation, and signal processing [3,4]. As clinical demand grows for more personalized, long-lasting, and bio-compliant devices, innovation in both materials and fabrication processes becomes essential.

Significant advances in neuroprosthetic technologies have improved performance, miniaturization, and biocompatibility [3]. However, conventional manufacturing techniques such as photolithography and the production of microelectromechanical systems (MEMS) have specific limitations, including the inability to handle soft, stretchable materials and difficulty in integrating multiple functional layers on non-planar substrates [5]. This has made it necessary to explore alternative advanced production methods that can improve the functionality and adaptability of neuroprosthetic devices [5,6].

The effectiveness of neuroprosthetic devices depends on their ability to seamlessly interface with biological tissues [6]. Key challenges in neuroprosthetic fabrication include achieving high-resolution features, ensuring biocompatibility, and integrating diverse materials with different mechanical and electrical properties [7]. Advanced manufacturing techniques, such as three-dimensional (3D) printing, laser-assisted printing, and aerosol jet printing (AJP), have emerged as promising solutions to overcome these challenges [8].

Among these techniques, AJP stands out due to its unique capability to deposit highly accurate patterns/traces of functional materials onto a variety of substrates. The non-contact AJP technique enables the designers to conceptualize complex structures with high spatial resolution, which is particularly advantageous for neuroprosthetic applications that require complex geometries and miniaturized components [9,10]. In neuroprosthetic device fabrication, AJP enables the realization of microelectrode arrays and neural interfaces with fine features [10]. It also supports the deposition of conductive, dielectric, and even bioactive materials, which is critical for the creation of multifunctional systems that combine sensing, stimulation, and data transmission on a single platform [8]. Furthermore, its compatibility with soft substrates allows the development of stretchable and conformable electronics. This is an essential requirement for devices that must be worn or implanted on curved and dynamic surfaces, such as skin, muscles, or neural tissue [11]. By leveraging these advantages, AJP has the potential to advance the design and manufacturing of neuroprosthetic devices, ultimately improving their performance and clinical applicability.

This review aims to provide the first comprehensive and application-specific analysis of AJP in the context of neuroprosthetic devices. While AJP has been reviewed broadly in flexible electronics [12,13], its role in neural interface technologies has not been systematically explored. We offer a detailed evaluation of AJP’s mechanisms, material compatibility, and patterning capabilities. This is presented alongside a critical comparison with conventional and emerging fabrication techniques, including inkjet, photolithography, MEMS, and laser-based methods. In doing so, we highlight how AJP enables conformal, multi-material, and biocompatible device fabrication uniquely suited to the anatomical and functional demands of neuroprosthetic devices. By identifying technical challenges, regulatory considerations, and future directions (e.g., biosensing integration, AI-enhanced systems, and clinical translation), this review serves as a practical resource to accelerate innovation and guide researchers in this rapidly advancing interdisciplinary field.

## 2. Principles of Aerosol Jet Printing (AJP)

### 2.1. Overview of AJP Technology

Originally developed by Optomec Inc. (Albuquerque, NM, USA) in 2007, aerosol jet printing (AJP) technology has gained prominence in the field of printed electronics due to its ability to deposit fine features with resolutions down to ~10 µm. This surpasses the typical resolution of inkjet printing (~20–50 µm) and provides a simpler, maskless alternative to traditional subtractive methods like photolithography and MEMS-based fabrication, which, while offering sub-micrometer resolution, require rigid substrates, cleanroom conditions, and complex multi-step processing [14,15]. In contrast, AJP enables the direct writing of conductive, dielectric, and bioactive materials from digital designs, supporting rapid prototyping and scalable production.

AJP stands out among additive manufacturing techniques for its non-contact deposition method, which uses a focused aerosol stream to pattern materials on a wide range of substrates, including curved ones. This allows AJP to overcome limitations of conventional techniques, which perform poorly on curved surfaces, but also from laser-based direct-write techniques such as laser-induced forward transfer (LIFT). While LIFT offers even higher resolution (<1 µm), it is typically limited by substrate constraints, optical material requirements, and potential thermal damage, all factors that are less problematic in AJP [16].

A critical advantage of AJP lies in its broad material compatibility. It supports ink viscosities ranging from 0.001 to 2.5 Pa·s, significantly exceeding the limits of inkjet systems and allowing the deposition of metallic nanoparticles, conductive polymers, ceramic-based inks, and biocompatible hydrogels [13,14]. Unlike photolithographic and MEMS-based approaches, which impose limitations due to etching, masking, or thermal steps, AJP is better suited for printing directly onto flexible, stretchable, and anatomically complex surfaces [15]. These attributes make AJP especially advantageous for developing next-generation neural interfaces, microelectrode arrays, and biosensors in neuroprosthetic applications [8,17]. A comprehensive comparison of different fabrication techniques relevant to neuroprosthetic applications is provided in Table 1.

#### 2.1.1. Mechanisms of Aerosol Generation and Deposition

**Figure 1:**
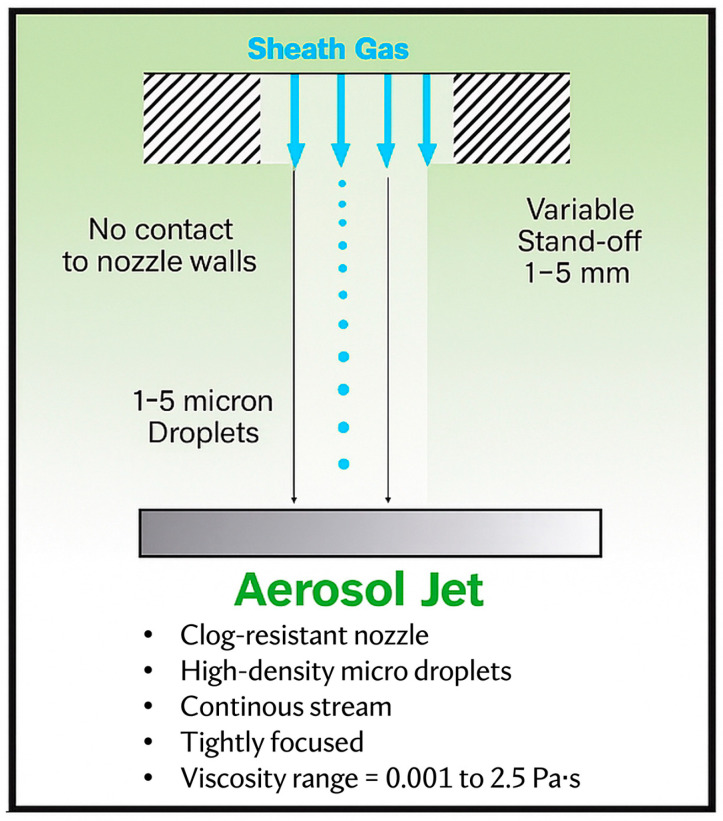
General principles of AJP technology. AJP uses sheath gas to focus aerosolized microdroplets into a tightly directed, non-contact stream. This enables printing with variable stand-off distances (1–5 mm), supports a wide viscosity range (0.001–1 Pa∙s), and reduces nozzle clogging. The system is compatible with high-density inks and flexible substrates, making it well-suited for printing complex, biocompatible structures on non-planar surfaces.

Unlike traditional printing techniques, such as inkjet or screen printing, which rely on direct liquid deposition, AJP operates by generating an aerosol of micron-sized liquid droplets, which is precisely focused into a collimated aerosol jet (Figure 1). This non-contact approach enables high-resolution patterning on a wide range of substrates with resolutions as fine as 10 µm and makes AJP particularly suitable for applications such as neural interfaces, microelectrode arrays, and flexible bioelectronic devices [10,14].

**Figure 2:**
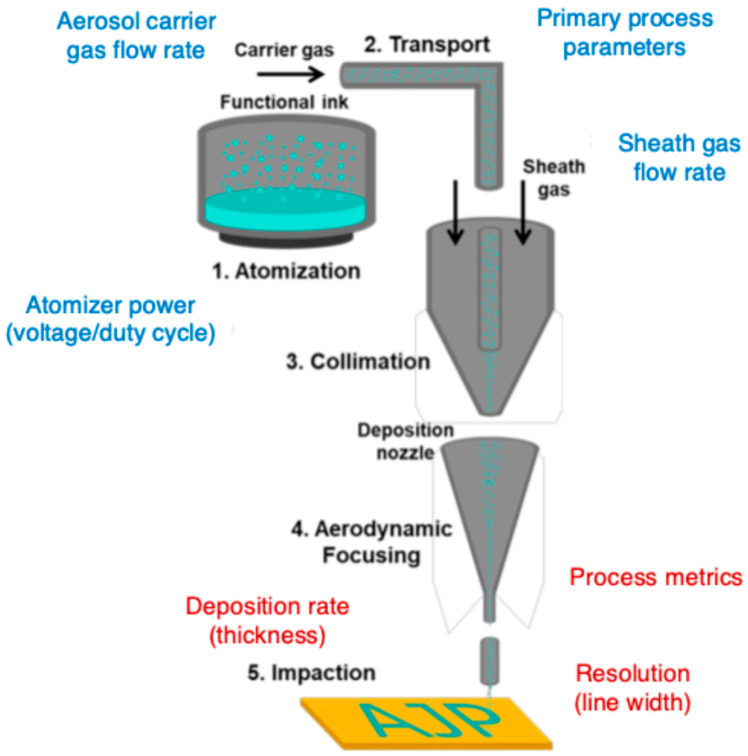
Schematic of aerosol jet printing process. The diagram illustrates five main stages: (1) atomization—liquid ink is converted into a fine mist using an ultrasonic or pneumatic atomizer; (2) transport—the aerosol is conveyed toward the nozzle via a carrier gas; (3) collimation—the aerosol stream is aligned and stabilized; (4) aerodynamic focusing—a sheath gas surrounds and narrows the stream to a micrometer-level beam; and (5) impaction—the aerosolized material is deposited onto the substrate in precise patterns. The figure is adapted from [14], with permission.

The AJP process is focused on the generation, transport, and accurate deposition of materials, ensuring controlled and reproducible ink efflux. This process involves three key steps: aerosol generation, aerosol transport and aerodynamic focusing, and deposition onto the substrate (Figure 2). Each step is meticulously optimized to maintain the stability of the aerosol stream and ensure uniform material deposition, which is crucial for applications in neuroprosthetic devices, where consistency and precision are essential [18].

#### 2.1.2. Aerosol Generation: Atomizing Functional Materials into Micron-Sized Droplets

The first step in the AJP process is converting liquid-phase ink into an aerosol, which is a suspension of fine droplets in a carrier gas. The formation of a well-controlled aerosol is vital, as the size and stability of the droplets significantly impact the quality of deposition. Ideally, the aerosol should contain droplets ranging from 1 to 5 µm in diameter to ensure that the deposited material forms continuous and well-defined structures upon impact with the substrate [8].

Aerosol generation is achieved through two primary mechanisms: ultrasonic atomization and pneumatic atomization [19,20]. Ultrasonic atomization uses high-frequency acoustic waves to induce cavitation in the atomizer. The oscillating pressure field generates capillary waves at the liquid surface, which eventually break apart into fine droplets [19]. This technique is particularly effective for inks with low-to-moderate viscosity, such as conductive polymers, biocompatible hydrogels, and enzymatic inks used in biosensors. Due to its ability to produce uniform droplet sizes, ultrasonic atomization is often preferred for applications requiring high-resolution patterning with minimal material wastage [21].

On the other hand, pneumatic atomization employs a high-speed gas stream (typically nitrogen or air) to shear the ink into fine droplets. It accommodates a wider range of ink viscosities, including metallic nanoparticle suspensions, but often results in a broader droplet size distribution [20]. Consequently, additional filtration or focusing techniques may be needed to refine the aerosol stream before deposition. Pneumatic atomization is commonly used for high-viscosity inks containing nanoparticles, such as silver, gold, or carbon-based conductive materials, which are essential for fabricating neural electrodes and high-performance interconnects in neuroprosthetic systems.

Regardless of the atomization method, the generated aerosol remains stable and well-dispersed to ensure uniform material delivery. Adding stabilizing agents or surfactants to the ink formulation can help prevent droplet coalescence and maintain consistent aerosol quality over prolonged printing durations [22,23].

#### 2.1.3. Aerosol Transport and Aerodynamic Focusing: High-Resolution Deposition

Once the aerosol mist is generated, it must be efficiently transported to the deposition head while preserving droplet integrity and uniformity. This is achieved using a carrier gas, typically nitrogen or air, which conveys the suspended droplets from the atomization chamber to the deposition nozzle. The rate of aerosol transport is a critical parameter, as it directly influences the printing resolution and uniformity of deposited features [24].

To achieve high-resolution patterning, AJP employs a unique aerodynamic focusing mechanism that refines the aerosol stream into a tightly collimated jet. This process is facilitated by a sheath gas flow, an additional stream of inert gas (such as nitrogen) that surrounds and compresses the aerosol stream, guiding it into a narrow, well-defined beam [14,25]. The sheath gas serves multiple functions, including collimation of the aerosol stream to ensure precise deposition and minimize unwanted material dispersion, acceleration of the aerosol particles to reduce their tendency to diffuse before reaching the substrate, and prevention of nozzle clogging by reducing particle accumulation at the nozzle exit.

The degree of aerosol focusing is governed by several key parameters. The sheath gas flow rate is crucial, as higher flow results in tighter beam focusing, leading to finer feature sizes. However, excessive sheath gas flow can cause over-constriction, reducing deposition efficiency. The achievable resolution is determined by the droplet size, while finer patterning is enabled by smaller nozzles (e.g., diameters of 100 µm or less), which may require precise ink formulation to prevent clogging [26,27]. Additionally, the carrier gas-to-sheath gas ratio must be carefully balanced to ensure optimal deposition control, preventing excessive material dispersion or undesired variations in line thickness [28].

Unlike inkjet printing, which requires the substrate to be positioned close to the print head, AJP allows for a stand-off distance of 1–5 mm, enabling deposition onto non-planar and flexible surfaces without physical interference. This feature is particularly beneficial for printing on implantable or wearable neuroprosthetic components, where surface curvature and flexibility are essential design considerations.

#### 2.1.4. Material Deposition: Achieving Precision in Neuroprosthetic Applications

The final stage in the AJP process is the deposition of aerosolized material onto the substrate, where the liquid droplets coalesce to form a continuous film or patterned structure. Unlike traditional inkjet methods, which rely on discrete droplet placement, AJP employs continuous material deposition. This approach can lead to uniform trace formation with reduced surface roughness under optimized conditions [29,30].

The deposition process depends on several interdependent factors [17]. Substrate properties, such as roughness, wettability, and temperature, can significantly influence print quality. Pre-treating substrates can improve material–substrate interactions. The deposition speed also plays a role; faster print speeds yield thinner layers, whereas slower speeds allow for greater material buildup. This tunability is essential when designing multilayered neural interfaces or microelectrode arrays that require precise thickness control.

### 2.2. Materials Used in AJP for Neuroprosthetic Applications

The efficiency and optimization of AJP for depositing diverse materials with high precision is advantageous for the development of neural interfaces, electrode arrays (capable of electrically interacting with each other), and flexible bioelectronic devices [8,11,31]. Unlike traditional fabrication techniques that impose significant constraints on material properties, AJP supports both metallic and polymeric inks, offering greater versatility in device design (Table 2).

In neuroprosthetic applications, the choice of materials is dictated not only by their electrical and mechanical properties but also by their biocompatibility and long-term stability [8,11]. The deposited materials must seamlessly integrate with biological tissues while maintaining functionality over extended periods. This necessitates careful consideration of conductive materials for signal transmission, materials for electrical insulation, and bioactive materials for enhanced tissue interaction.

#### 2.2.1. Conductive Materials for Neural Interfaces and Bioelectronic Devices

Conductive materials play a pivotal role in neuroprosthetic devices, as they form the fundamental components of electrodes, interconnects, and sensing elements. AJP enables the precise deposition of several classes of conductive materials, each offering distinct advantages depending on the intended application.

Metallic nanoparticle inks, particularly silver, gold, platinum, and copper, are commonly used due to their excellent electrical conductivity [32,33]. Silver and gold, in particular, have been widely employed in neuroelectrode fabrication due to their high corrosion resistance and stable electrochemical properties. Platinum is one of the materials that can be used for long-term implantable devices, as it demonstrates superior biocompatibility and can withstand the harsh physiological environment. However, despite their high conductivity, metallic inks often require post-processing treatments, such as thermal sintering or laser curing, to enhance their structural integrity and adhesion to flexible substrates. This can present challenges when working with temperature-sensitive materials, necessitating the use of low-temperature sintering techniques or alternative conductive materials.

Beyond metals, carbon-based materials, such as carbon nanotubes (CNTs) and graphene, have garnered increasing interest for their flexibility, mechanical robustness, and biocompatibility [34,35]. CNT-based inks offer the advantage of high conductivity with mechanical compliance, making them ideal for stretchable and flexible neuroprosthetic applications. Moreover, CNTs and graphene exhibit low impedance at neural interfaces, improving signal transduction efficiency while minimizing unwanted electrochemical reactions [36,37].

Another emerging class of materials in neuroprosthetic applications is conductive polymers, such as poly(3,4-ethylenedioxythiophene) polystyrene sulfonate (PEDOT:PSS) [38,39]. These polymers offer a special combination of electrical conductivity, mechanical flexibility, and biofunctionality, making them well-suited for neural recording and stimulation electrodes. Unlike metal-based conductors, PEDOT:PSS can be deposited at room temperature without additional sintering, allowing for greater compatibility with soft, organic substrates. Additionally, conductive polymers can be chemically engineered to enhance cell adhesion or promote neural growth, further expanding their applicability in bioelectronic medicine.

#### 2.2.2. Dielectric and Biocompatible Polymers for Insulation and Encapsulation

In neuroprosthetic fabrication, insulating and protective layers are equally as important as conductive elements. These materials serve to electrically isolate different components, enhance mechanical durability, and provide biocompatibility for implantable systems [40].

Polyimides and silicones have long been favored in bioelectronic applications due to their thermal stability, mechanical flexibility, and excellent insulating properties [41]. Polyimides, in particular, are widely used as substrates for flexible neural interfaces, as they can be printed in thin layers while maintaining high mechanical strength. AJP enables the direct deposition of these materials with micron-scale precision, facilitating the fabrication of multi-layered devices with integrated conductive and insulating components.

Similarly, silicone-based materials are frequently employed as encapsulation layers for implantable neuroprosthetic devices. Their ability to conform to soft tissues while providing long-term biostability makes them an ideal choice for devices requiring prolonged implantation. Importantly, silicones are compatible with low-temperature post-processing techniques used in AJP, such as UV curing or mild thermal annealing, which helps preserve the mechanical integrity of flexible substrates [41]. Moreover, recent advances in AJP have enabled the deposition of elastomeric materials, paving the way for the development of stretchable bioelectronics that can adapt to natural tissue movements without compromising functionality [42,43].

Another category of insulating materials used in AJP-based neuroprosthetic devices is Parylene coatings, which offer excellent moisture resistance and chemical stability [44]. Parylene is particularly valuable for protecting sensitive electronic components from bodily fluids, ensuring long-term device reliability. Since AJP allows for the localized deposition of such coatings, it provides a more efficient and controlled alternative to conventional dip-coating or vapor deposition techniques.

#### 2.2.3. Functional Inks for Biointegrated Neuroprosthetic Systems

Beyond conventional conductors and insulators, the next generation of neuroprosthetic devices increasingly relies on functional inks with biointeractive properties [45]. These advanced materials promote electrical signal transmission but also take an active role in tissue integration, cellular communication, and real-time physiological monitoring. A particularly promising class of functional materials in neuroprosthetic devices is bioactive and biomimetic inks [46,47]. These inks incorporate neurotrophic factors, adhesion peptides, or extracellular matrix components that support cell adhesion, growth, and differentiation. By harnessing AJP’s precise spatial control, biochemical gradients can be printed to guide neural regeneration, ultimately enhancing the performance of implantable interfaces in restoring motor and sensory functions. In addition to bioactive coatings, AJP has been increasingly explored for the deposition of piezoelectric and magnetostrictive materials that enable real-time sensing and actuation in neuroprosthetic applications. Piezoelectric polymers, such as polyvinylidene fluoride (PVDF), can convert mechanical deformations into electrical signals, enabling self-powered sensing in smart prosthetics [48,49]. Similarly, magnetostrictive materials have been investigated for wireless neural stimulation systems, where externally applied magnetic fields induce electrical currents within the implanted device [50]. These materials mark a shift in neuroprosthetic design, from traditional passive electrodes to bioelectronic systems that actively respond to external stimuli.

Another exciting avenue of research involves the integration of biosensing materials within neuroprosthetic platforms. AJP allows for the precise patterning of enzymatic or electrochemical biosensors onto flexible substrates, enabling continuous monitoring of neurochemical activity, glucose levels, or inflammatory markers [51]. This capability is particularly relevant for next-generation closed-loop neuroprosthetic systems, where real-time feedback can be used to dynamically adjust stimulation parameters based on the patient’s physiological state. While most AJP-fabricated biosensors have so far been validated in benchtop or wearable human studies (e.g., EMG monitoring, pressure sensing), their application in preclinical animal models for neuroprosthetic use remains an important next step toward clinical translation.

The adaptability of AJP to print multi-material architecture further enhances its potential in neuroprosthetic device development. By seamlessly integrating conductive, dielectric, bioactive, and sensing materials into a single fabrication process, AJP facilitates the development of highly functional, miniaturized, and patient-specific neural interfaces. The ability to customize material compositions at the microscale opens up new possibilities for tailoring neuroprosthetic devices to individual patient needs, ultimately advancing the field toward more effective and personalized neural rehabilitation technologies.

## 3. Applications of AJP in Neuroprosthetic Devices

### 3.1. Fabrication of Neural Interfaces

The advancement of neuroprosthetic devices relies heavily on the development of high-density neural interfaces capable of accurately recording and stimulating neural activity. AJP has emerged as a transformative fabrication method for these interfaces, enabling the creation of customized neural probes tailored to specific brain regions [8,17]. Unlike conventional manufacturing techniques, which often require complex lithographic processes, AJP provides a streamlined, mask-free approach that allows for the rapid prototyping and scalable production of neural interfaces.

A key advantage of AJP in neural probe fabrication is its ability to deposit fine, conductive traces, with resolutions down to 10 μm [52]. This precision is crucial for developing electrodes that can detect weak neural signals while minimizing tissue damage [53]. For instance, researchers have successfully printed platinum-based microelectrodes on flexible polyimide substrates using AJP, resulting in probes with enhanced signal resolution and durability [11].

Additionally, AJP supports the deposition of biocompatible coatings, such as polyimides and PEDOT:PSS, which enhances implant longevity and functionality [38]. In deep brain stimulation, PEDOT:PSS-coated electrodes have demonstrated improved charge transfer and reduced impedance, potentially improving treatment outcomes for conditions like Parkinson’s disease [54].

Another key application of AJP-fabricated interfaces is in brain–computer interfaces (BCIs), which convert neural activity into digital commands, enabling control of prosthetic limbs or external devices by individuals with severe motor impairments [40]. For example, a study demonstrated enhanced signal acquisition in BCIs by integrating AJP-printed silver-nanoparticle traces with flexible graphene sensors, leading to greater accuracy in real-time robotic control [55].

AJP’s fine-resolution capabilities, material compatibility, and ability to conform to soft tissues make it a promising platform not only for research-grade neural probes but also for clinical systems. These include cochlear implants and bionic prostheses, where conformable AJP-printed microelectrode arrays and sensors could improve signal fidelity, device integration, and user comfort [56,57,58]. The integration of AJP-fabricated components into such systems may accelerate clinical translation by enabling efficient, customizable, and scalable manufacturing.

### 3.2. Development of Flexible and Stretchable Electronics

Neuroprosthetic devices must accommodate natural body movements, often requiring electronics that are both flexible and stretchable. Traditional rigid components are unsuitable for applications involving dynamic motion, which has led to increased interest in AJP for its ability to print directly onto elastomers and ultra-thin polymers [42,43].

Wearable neuroprosthetic devices, such as soft neural sensors and bioelectronic skins, benefit from AJP-printed stretchable conductive traces that maintain electrical performance under mechanical strain. For instance, AJP was used to print silver-nanowire networks onto ultra-thin thermoplastic polyurethane, resulting in a durable, high-sensitivity skin-integrated electrophysiological monitoring system [59].

This combination of biocompatible materials and customizable sensor architectures supports prolonged device wear and accurate physiological monitoring, including muscle activity, heart rate, and neural responses [60]. AJP-fabricated stretchable sEMG sensors have also been successfully integrated into smart exoskeletons, enhancing rehabilitation technologies [61].

AJP’s compatibility with soft substrates has also enabled the development of implantable neural interfaces that adapt to the spinal cord and brain without imposing mechanical stress on surrounding tissues. For example, ultra-thin, stretchable electrode arrays for spinal cord stimulation have been fabricated using AJP [62].

In addition to mechanical flexibility, AJP offers unprecedented anatomical personalization. Maskless digital printing allows devices to be tailored to individual morphologies, such as cranial curvatures or residual limbs, enhancing both comfort and interface quality [9,48,63]. Personalized electrode and sensor configurations can align more precisely with patient-specific anatomical and functional characteristics, improving signal fidelity, control precision, and sensory feedback. This, in turn, can promote cortical reorganization and motor learning, leading to more effective rehabilitation outcomes and better long-term user adaptation.

### 3.3. Creation of Microelectrode Arrays

**Figure 3:**
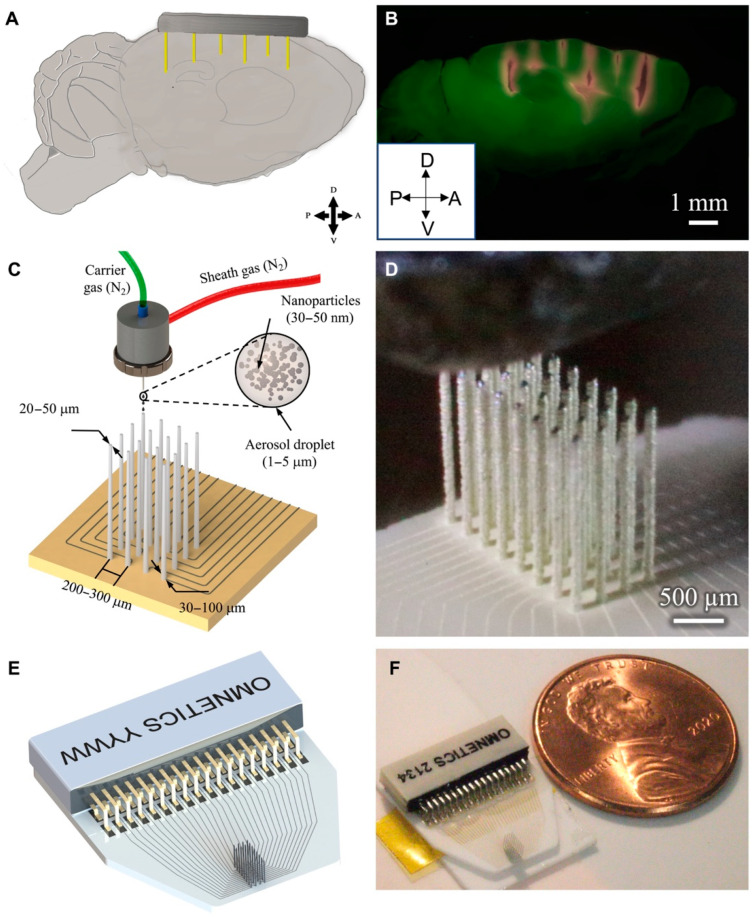
Example of AJP printing process for neural probes. (**A**) Conceptual schematic targeting mouse brain regions: prefrontal cortex, motor cortex, caudal striatum, and hippocampal CA2/CA3. (**B**) Sagittal brain slice showing 3D-printed probe shanks (red) penetrating cortex, striatum, and hippocampus. (**C**) Schematic of rapid AJP-based 3D printing of metal nanoparticles for customizable neural probes and circuit routing. (**D**) Image of a 32-channel probe during printing. (**E**) Schematic design of the probe with routing and connector. (**F**) Final printed probe next to a U.S. one-cent coin for scale. This figure is reproduced from [53], with permission.

Microelectrode arrays (MEAs) are a cornerstone of neuroprosthetic applications, providing precise neural recording and stimulation capabilities. These arrays must be designed with high electrode densities to achieve fine spatial resolution while maintaining minimal invasiveness [38,44]. The ability of AJP to print micron-scale conductive pathways enables the fabrication of customized MEAs with variable electrode configurations (Figure 3), optimizing their performance for specific applications [64].

Building on the high-resolution and biocompatibility advantages discussed in Section 3.1, AJP allows for high-density electrode patterning and the deposition of low-impedance coatings such as PEDOT:PSS [65,66]. These features enhance signal fidelity and electrode–tissue interactions, which are critical for reliable, long-term use in both recording and stimulation contexts.

As described in Section 3.2, AJP also supports the fabrication of flexible MEA configurations that conform to diverse anatomical structures, including cortical folds and spinal surfaces [41,67]. This adaptability enables clinicians to target specific neural regions more effectively, enhancing the performance and user comfort of neuroprosthetic systems.

In addition, AJP enables the integration of functional materials, such as conductive polymers and bioactive coatings, that promote neural adhesion and biocompatibility [10,68]. These features support stable electrode–neuron interfaces and are particularly valuable in neural rehabilitation therapies where MEAs are used to re-engage damaged neural circuits. Enhancing this interface can contribute to a more effective restoration of motor and sensory functions in patients.

### 3.4. Integration with Other Fabrication Technologies

The versatility of AJP extends beyond standalone applications, as it can be seamlessly integrated with other advanced fabrication techniques to create hybrid neuroprosthetic devices. By combining AJP with 3D printing, lithography, or microfabrication methods, researchers can design multi-functional neuroprosthetic systems that incorporate microfluidic components, biosensors, and soft robotics into a single, cohesive platform [21,69,70].

For instance, integrating AJP with 3D printing technologies enables the fabrication of complex, multi-layered devices with embedded electronics. This approach is particularly useful for developing smart neuroprosthetic devices that incorporate self-sensing capabilities, adaptive stimulation, and real-time signal processing. The synergy between AJP and 3D printing allows for the creation of complex structures that can perform multiple functions, enhancing the overall effectiveness of the neuroprosthetic devices [40].

Similarly, AJP can be used alongside lithographic patterning techniques to refine the resolution of printed structures, ensuring compatibility with high-performance bioelectronic applications [18]. This integration allows for the precise positioning of conductive pathways and other critical components, which is essential for the development of advanced neural interfaces and sensors.

The hybridization of AJP with other fabrication techniques not only expands the design possibilities for neuroprosthetic devices but also significantly enhances their overall functionality. By leveraging multiple material deposition strategies, researchers can create next-generation neural implants that offer superior biocompatibility, electrical performance, and mechanical resilience. This multi-faceted approach enables the development of neuroprosthetic devices that are more robust, reliable, and capable of meeting the complex demands of neural rehabilitation and other medical applications [12,71].

## 4. Shortcomings and Challenges

### 4.1. Technical Challenges in AJP for Neuroprosthetic Devices

Despite the numerous advantages of AJP in neuroprosthetic fabrication, several technical challenges must be addressed to realize its potential. One of the primary difficulties lies in maintaining high resolution and accuracy, particularly when printing complex neural interfaces with complex designs [26,27]. The ability to deposit fine conductive pathways with uniform dimensions is essential for reliable neural stimulation and signal acquisition [72]. However, variations in aerosol flow dynamics, substrate interactions, and material deposition rates can lead to inconsistencies in printed features, which can compromise the functionality of the neuroprosthetic devices.

Another significant challenge is ensuring process repeatability and process scalability for large-scale manufacturing [73,74]. Small deviations in printing parameters, such as carrier gas flow rate, ink viscosity, and substrate alignment, can introduce defects that compromise device performance. To address these challenges, advanced real-time monitoring and adaptive control systems are being developed. These include machine-vision-based feedback loops and in situ process sensors that enhance the precision and reproducibility of AJP-based neuroprosthetic device fabrication [25]. By maintaining consistent printing conditions, such systems help reduce defects and improve overall device quality.

### 4.2. Material Compatibility and Biocompatibility

Material selection is a critical aspect of neuroprosthetic development, as the materials used must not only fulfill functional and electrical requirements but also meet strict biocompatibility standards. Neuroprosthetic implants interact directly with biological tissues, necessitating materials that do not provoke inflammatory responses or degrade over time [75]. Many traditional electronic materials, such as silver and copper, pose biocompatibility challenges, requiring protective encapsulation or alternative material solutions to ensure safe and effective use in the body.

Additionally, printed components must resist degradation due to factors such as moisture, enzymatic activity, and mechanical stress [61]. Advanced biofunctional materials, such as PEDOT:PSS and graphene-based materials, are being explored to enhance both electrical conductivity and tissue integration [34,38]. These coatings can improve the performance and longevity of neuroprosthetic devices by providing a stable interface between the device and the biological tissue. Further research is needed to identify and optimize materials that offer an ideal balance of conductivity, flexibility, and biocompatibility, ensuring that the devices remain functional and safe over extended periods.

### 4.3. Reliability and Long-Term Stability of Printed Devices

For neuroprosthetic devices to be clinically viable, they must demonstrate long-term durability and reliability under real-world conditions. Printed electrodes and interconnects must maintain their conductive properties without delaminating, cracking, or experiencing signal drift over time.

To enhance the long-term stability of AJP-printed neuroprosthetic devices, several strategies can be employed. Optimized ink formulations can improve adhesion and mechanical flexibility, ensuring that the printed components remain intact and functional despite the stresses they encounter [76]. Protective encapsulation layers can prevent degradation from bodily fluids, shielding the sensitive electronic components from the harsh physiological environment [77]. Rigorous mechanical testing replicates real-world usage conditions to assess fatigue resistance, identify potential failure points, and guide improvements in overall device durability.

Ensuring that printed neuroprosthetic devices remain functional over extended periods is crucial for their clinical adoption and patient safety [11]. By addressing these challenges and continuously improving the materials and processes used in AJP, researchers can develop more reliable and effective neuroprosthetic devices that enhance the quality of life for patients with neurological conditions.

## 5. Emerging Developments in AJP-Based Neuroprosthetic Devices

### 5.1. Technological Advances in AJP

Recent developments are significantly enhancing the capabilities of aerosol jet printing (AJP) for neuroprosthetic device fabrication. These advances aim to improve spatial resolution, reliability, and adaptability to complex anatomical surfaces, critical requirements for implantable and wearable neural technologies. Hybrid approaches combining AJP with laser direct writing have demonstrated enhanced patterning resolution and improved compatibility with curved substrates, making them especially promising for complex bioelectronic structures [78,79]. Recent studies on ultrasonic atomizers have also advanced understanding of mist formation and droplet control. For example, a lumped-parameter model was developed to describe mist behavior in ultrasonic atomizers, enabling better control over aerosol saturation and droplet uniformity. This is crucial for stable deposition on curved or non-planar substrates used in neural interfaces [80]. Complementing atomizer advances, recent computational fluid dynamics models have provided deeper insights into the influence of nozzle geometry, ink properties, and sheath gas flow on aerosol stream behavior, helping to improve line resolution and repeatability [20,25,26]. Furthermore, emerging real-time monitoring and closed-loop control systems are further improving the reliability of AJP for biomedical applications. A diagnostic method using light scattering was developed to monitor aerosol deposition rate, enabling real-time correction of process parameters [81,82]. These capabilities are especially important for neuroprosthetic devices, where high spatial precision and consistency are essential across soft or patient-specific substrates.

### 5.2. Realization of Feedback Restoration in Bionic Limbs

**Figure 4:**
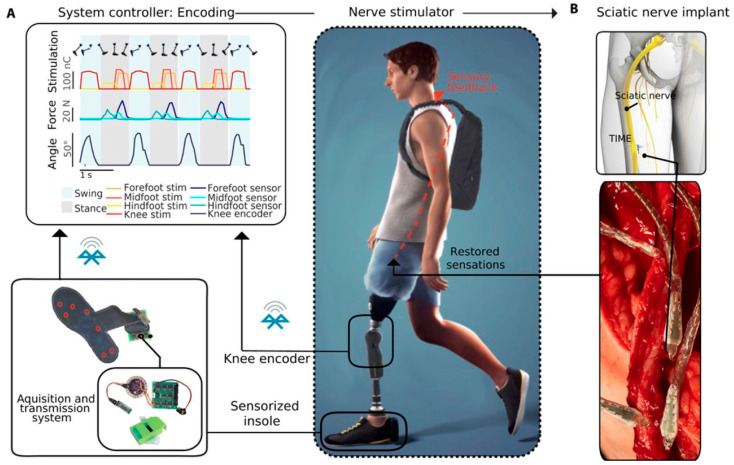
Neuroprosthetic leg. A lower limb amputee wears a custom-made prosthesis with knee and ankle components equipped for sensory feedback. An encoder in the prosthetic knee (**A**) measures flexion, and a sensorized insole under the foot collects pressure data. Sensor outputs are transmitted to an external controller that activates a stimulator connected to transversal intrafascicular multichannel electrodes implanted in the tibial portion of the sciatic nerve (**B**), with neural interfaces placed within the nerve fascicles. This figure is reproduced from [86], with permission.

One of the most transformative future directions in neuroprosthetic device development is the feedback restoration in prosthetic limbs, which typically transform data coming from force/pressure and position sensors into tactile and proprioceptive feedback (Figure 4). Current prosthetic limbs offer limited sensation, making fine motor control challenging [83]. AJP facilitates the fabrication and integration of high-density, flexible sensor networks that can be embedded in prosthetic surfaces. Recent prototypes have demonstrated the feasibility of integrating aerosol-printed sensors, such as stretchable EMG electrodes and pressure-sensitive layers, directly onto wearable prosthetic systems [59,61]. The integration of real-time sensory feedback enhances motor coordination and adaptability, providing users with a more natural experience [84]. Furthermore, advancements in machine-learning algorithms enable these prosthetics to dynamically adjust their response based on sensory input, improving precision and usability [85]. AJP’s ability to print customizable, highly responsive sensor arrays makes it a valuable tool in next-generation prosthetic technology.

### 5.3. Integration of Biosensors for Monitoring Physiological Parameters

The incorporation of biosensors into neuroprosthetic devices represents another groundbreaking innovation. Biosensors can detect key physiological parameters, such as neural activity, glucose levels, oxygen saturation, and muscle contractions. This enables critical real-time health monitoring [87]. AJP plays a pivotal role in this development by enabling the fabrication of miniaturized, high-resolution biosensors with flexible form factors [51]. These sensors can be integrated into neuroprosthetic systems to provide continuous data feedback to users and healthcare providers [88]. This technology is particularly beneficial for individuals with chronic conditions like diabetes or neurological disorders. It supports early detection of complications and enhances medical management [3]. By continuously monitoring physiological signals, biosensors help maintain optimal health and prevent issues before they become severe.

### 5.4. Control of Bionic Limbs

Neuroprosthetic devices are evolving toward smart prosthetic limbs that incorporate real-time data processing. These systems utilize artificial intelligence (AI) and machine learning to analyze high-density, often multimodal, sensory inputs. This enables prosthetic movements to be optimized based on user behavior and environmental conditions. AJP supports this innovation by allowing precise fabrication of customized circuits that connect to high-density sensor arrays—key components of adaptive, self-learning prosthetic limbs. These advanced devices can recognize and adapt to user movement patterns. They enhance response time using predictive control and improve over time through AI-based learning algorithms [89]. The result is a shift from static devices to dynamic, user-responsive prosthetics with improved mobility and function. These systems promise a more intuitive experience, adapting in real time to each user’s needs.

### 5.5. Prospects for Clinical Translation and Commercialization

While AJP-based devices have not yet reached clinical trials, preclinical studies have demonstrated promising results. Specifically, AJP has been used to create biocompatible neural interfaces and wearable biosensors. These include EMG electrodes and PEDOT:PSS-based platforms designed for neural signal recording [10,61]. These developments underscore AJP’s viability for producing complex, conformal electronics tailored to biological tissues. However, the path from laboratory innovation to clinical application presents several regulatory and commercialization challenges. Ensuring that AJP-based neuroprosthetic devices meet the stringent safety and efficacy standards of regulatory bodies, such as the U.S. Food and Drug Administration (FDA) and the European Medicines Agency (EMA), is essential for widespread clinical adoption. Key factors influencing the successful commercialization of AJP-based neuroprosthetic devices include regulatory compliance, the establishment of standardized testing protocols for long-term safety and reliability, and the development of scalable, cost-effective manufacturing processes. Continued technological maturation and interdisciplinary collaboration will be essential to move AJP-enabled devices closer to clinical translation. Additionally, reducing production costs is crucial to making these neuroprosthetic devices affordable and accessible to a broader population. Due to the increasing demand for advanced prosthetic solutions, AJP-based neuroprosthetic devices hold strong market potential. Continued investment in research, development, and translational efforts will be crucial in bringing these cutting-edge technologies to clinical reality, ultimately improving the quality of life for individuals with disabilities and neurological impairments.

## 6. Conclusions

In summary, AJP technology has emerged as a transformative tool for neuroprosthetic device development, offering unparalleled precision and versatility in fabricating complex, miniaturized structures essential for advanced neural interfaces. The foundational principles of AJP, including its unique mechanisms of aerosol generation and precise deposition, facilitate the use of diverse materials on various substrates. AJP is well suited for neuroprosthetic applications due to its scalability, ability to print on flexible and stretchable substrates, and compatibility with a wide range of biocompatible materials. Key applications include the fabrication of neural interfaces, the development of microelectrode arrays, and integration with flexible electronic systems.

The application of AJP for neuroprosthetic device development is promising, with numerous opportunities for innovation and improvement. The realization of sensorized prosthetic limbs, the integration of biosensors for monitoring physiological parameters, and the development of smart prosthetics with real-time data processing capabilities are just a few of the exciting directions for this technology. Continued research and development in AJP can drive significant progress in neuroprosthetic applications, leading to the creation of more effective and reliable devices. The prospects for clinical translation and commercialization are promising, with the potential to improve patient outcomes and expand access to advanced prosthetic technologies.

## Figures and Tables

**Table 1:** Comparison of fabrication techniques for neuroprosthetic devices, for resolution, material compatibility, flexibility, biocompatibility, and representative manufacturers.

Technique	Resolution	Material Compatibility	Flexibility	Biocompatibility	Manufacturers
Aerosol Jet Printing	~10 µm	Conductive inks, polymers, carbon-based materials, hydrogels	High (prints on flexible/stretchable substrates)	High (supports biocompatible materials)	Optomec, Neotech AMT
Inkjet Printing	~20–50 µm	Limited to low-viscosity inks, fewer biocompatible materials	Moderate (mostly for flexible electronics)	Moderate (some conductive inks need post-treatment)	Dimatix (Fujifilm), MicroFab
Photolithography	<1 µm	Metals, semiconductors, dielectrics	Low (requires rigid substrates)	High (if using biocompatible coatings)	SUSS MicroTec, EV Group
MEMS-based methods	<1 µm	Silicon-based materials, metals, polymers	Low (mainly rigid structures)	High (used in implantable neuroprosthetic devices)	Bosch, IMEC (custom foundries)
Laser-based printing	<1 µm	Optically responsive inks, limited bioinks	Low to moderate (mostly flat surfaces)	Variable (risk of thermal damage)	Luxexcel, InnoLaser, LPKF

**Table 2:** Overview of key material categories used in AJP for neuroprosthetic device applications. The table lists representative materials and summarizes their functional roles in enhancing neural interfaces and bioelectronic systems. These include conductive materials for signal transmission, dielectric and biocompatible polymers for structural support and encapsulation, and functional inks for sensing, stimulation, and tissue integration.

Material Category	Examples	Function in Neuroprosthetic Devices
Conductive materials	Silver, gold, platinum, copper	High conductivity, corrosion resistance, used in neuroelectrodes and interconnects
	Carbon nanotubes (CNTs), graphene	High flexibility, low impedance, improved signal transduction
	PEDOT:PSS (Conductive polymers)	Biocompatible, flexible, used for neural recording and stimulation
Dielectric & biocompatible polymers	Polyimides	Flexible substrates for neural interfaces
	Silicones	Encapsulation layers, long-term biostability
	Parylene	Moisture-resistant coating, chemical stability for implantable devices
Functional inks	Bioactive/biomimetic Inks	Promote neural adhesion, growth, and differentiation
	Piezoelectric polymers (PVDF)	Convert mechanical input into electrical signals or vice versa for sensing applications
	Magnetostrictive materials	Enable wireless neural stimulation using magnetic fields
	Biosensing materials	Monitor neurochemical activity, glucose levels, and inflammation for real-time feedback

## Abbreviations

The following abbreviations are used in this manuscript:

AI	Artificial Intelligence
AJP	Aerosol Jet Printing
BCI	Brain–Computer Interface
CNT	Carbon Nanotube
EMA	European Medicines Agency
EMG	Electromyography
FDA	Food and Drug Administration
MEA	Microelectrode Array
MEMS	Microelectromechanical Systems
PEDOT:PSS	Poly(3,4-ethylenedioxythiophene) Polystyrene Sulfonate
PVDF	Polyvinylidene Fluoride
sEMG	Surface Electromyography
